# Creating and Exploring Semantic Annotation for Behaviour Analysis

**DOI:** 10.3390/s18092778

**Published:** 2018-08-23

**Authors:** Kristina Yordanova, Frank Krüger

**Affiliations:** 1 Institute of Computer Science, University of Rostock, 18059 Rostock, Germany; 2 Institute of Communications Engineering, University of Rostock, 18119 Rostock, Germany; frank.krueger@uni-rostock.de

**Keywords:** semantic annotation, model-based, activity recognition, behaviour analysis

## Abstract

Providing ground truth is essential for activity recognition and behaviour analysis as it is needed for providing training data in methods of supervised learning, for providing context information for knowledge-based methods, and for quantifying the recognition performance. Semantic annotation extends simple symbolic labelling by assigning semantic meaning to the label, enabling further reasoning. In this paper, we present a novel approach to semantic annotation by means of plan operators. We provide a step by step description of the workflow to manually creating the ground truth annotation. To validate our approach, we create semantic annotation of the Carnegie Mellon University (CMU) grand challenge dataset, which is often cited, but, due to missing and incomplete annotation, almost never used. We show that it is possible to derive hidden properties, behavioural routines, and changes in initial and goal conditions in the annotated dataset. We evaluate the quality of the annotation by calculating the interrater reliability between two annotators who labelled the dataset. The results show very good overlapping (Cohen’s κ of 0.8) between the annotators. The produced annotation and the semantic models are publicly available, in order to enable further usage of the CMU grand challenge dataset.

## 1. Introduction

The annotation of sensor datasets describing human behaviour plays an important role in any system aiming to analyse the user behaviour. Annotation is essential both in designing and training the system to recognise and reason about the observed behaviour, either through the definition of a suitable symbolic model in knowledge-driven applications [1,2,3], or through the preparation of training data for learning tasks in data-driven models [4]. For that reason, the quality of annotation can have a significant impact on the performance of the derived systems. Annotation is also vital for validating and quantifying the performance of applications that aim at recognising the user behaviour. With intelligent systems relying increasingly on large datasets for designing and testing models of users’ activities, the process of data annotation is becoming a major concern for the community [5].

*Labelling* is a manual process, often done by analysing a separately recorded log (video or diary) of the conducted trial. It provides a target label for each observation in the sensor dataset based on the observed in the video log behaviour. The resulting sequence of labels is called *annotation*. It represents the underlying meaning of data [6] and provides a symbolic representation of the sequence of events.

While social sciences have a long history in annotating (coding) human behaviour [6], annotating sensor datasets in the ubiquitous computing community to provide the ground truth for sensor-based systems is a challenging and often unclear process [7]. It is usually the case that annotation processes are not described in detail and their quality is not evaluated, thus often making publicly available datasets and their provided annotations unusable [8]. In addition, most public datasets provide only textual labels without any semantic meaning. Thus, they are unsuitable for evaluating ubiquitous approaches that reason beyond the event’s class and are able to provide semantic meaning to the observed data [1].

To address the above problems, in this paper, we present an extended version of a model-based approach to semantic annotation of human behaviour based on the annotation process proposed in [9] and first presented in [10]. Furthermore, in this work, we provide the first evidence that the model-based approach can be used to reason beyond the action label. We show that the approach is able to infer contextual properties about the executed actions and user situation, which are not explicitly annotated but which can be reasoned about based on the underlying semantic structure. This is a novelty in the field of activity recognition, as current approaches to annotation provide simply a textual label to the data and do not allow any inference. In our approach, the labels assigned to the data provide an underlying semantic structure that contains information about the actions, goals, and plans being executed. This semantic structure is represented in the form of a model of the behaviour’s state in terms of collection of state variables. Actions are then defined as effects that change the state of the model. This form of annotation provides structured knowledge of the concepts in the data being annotated and enables the reasoning over underlying behaviour changes, their causal relations, and contextual dependencies. Such annotation is important for evaluating plan recognition approaches that aim not only to recognise the goal of the plan, but also the subgoals and actions being executed. Furthermore, the model-based semantic annotation is important for evaluating the performance of any approach that aims at recognising the underlying actions’ context. Finally, the annotation will be beneficial for approaches that strive to learn models of human behaviour.

The contribution of this paper is as follows: 1. we introduce a more detailed version of a novel approach to semantic annotation by means of precondition and effect rules; 2. we describe a step by step workflow to create such annotation; 3. we propose a method for improving the quality of the annotation through training of the annotators; 4. we provide a semantic annotation and the corresponding models for three types of recipes from the CMU grand challenge dataset; and 5. we show that the annotation allows for reasoning about hidden properties, context information and behaviour routines, which is a novelty in the field of annotation for activity recognition.

The paper is structured as follows: in Section 2, we discuss the types of annotation available in the literature and outline how our approach distinguishes from them. Section 3 describes the proposed approach, the corresponding workflow as well as strategies for improving the quality of the annotation by providing an initial training phase. Section 4 illustrates the approach by re-annotating the Carnegie Mellon University Multi-Modal Activity Database (CMU-MMAC). The new annotation together with the corresponding models is publicly available. We show that it is possible to reason about hidden properties, changes in the environment and behavioural patterns based on the underlying model. We evaluate our approach by calculating the interrater reliability between different annotators. Section 5 discusses the applicability of the approach to other problem domains. Finally, the paper concludes with a short discussion of the approach and the future work.

## 2. Annotation of Human Behaviour

Annotation is a process that has long been established in social sciences. Labelling (or coding) is usually applied on language-based or textual data there following a well established procedure [6]. According to Saldana [6], a code *“is most often a word or short phrase that symbolically assigns a summative, salient, essence-capturing, and/or evocative attribute for a portion of language-based or visual data.”* The result of coding is a sequence of labels (traces) that represents the underlying meaning of a written, spoken, or visual data. Coding is often used to produce behaviour traces that allow the measuring and analysis of human behaviour, for example in determining the progression of dementia [11].

Coding in social sciences usually relies on an underlying semantic structure in the form of ontology or taxonomy. This ontology groups the codes (labels) into different categories that represent the concepts describing the encoded behaviour. The ontology (or codebook) can be provided before the coding begins, or it could be developed/extended during coding [6].

A similar approach is followed when annotating data for recommender systems in web platforms. A folksonomy is developed there, based on which the annotator is provided with suggestions about suitable labels [12,13]. The folksonomy is then a codebook collaboratively developed by a group of people that annotate a given web resource. Here too, the idea is that, given a codebook, the annotators are able to assign tags or labels to given parts of the data.

The same principle is also used in sensor-based behaviour recognition approaches. The objective there is to assign a label to each time step of a sensor dataset describing human behaviour [14]. This is often done by analysing a separately recorded video log of the conducted trial. The resulting labels provide a symbolic representation of the true sequence of activities. Here too, the codebook can be developed by experts or collaboratively by a group of users.

### 2.1. Types of Annotation Based on Logging Method

While annotation in social sciences is mainly manually done, in the fields of ubiquitous computing and sensor-based behaviour analysis, we distinguish between manual, semi-automated, and automated methods for producing annotation [14]. Furthermore, the annotation can be done either online (parallel with recording the data) or offline (after the data is recorded) [14].

Manual annotation can be produced in three ways: 1. by observing a video log of the recorded behaviour [10,15]. This allows for producing very precise high quality annotation as the annotator can go back and re-annotate problematic parts of the log; 2. by directly observing the experiment participant and manually labelling their behaviour [16]. This allows for producing annotation on the run, which saves time, but, at the same time, produces annotation with relatively low reliability; 3. by self-annotating one’s own behaviour during the experiment [17,18,19]. This reduces the costs for external observer or offline annotation based on video logs. It, however, suffers from low annotation quality as the person is often unable to accurately annotate their activities on the run.

To address the problems of manual annotation, there are attempts at semi-automatically assigning tags to the sensor data [20]. The advantage in such approaches is that they require a small manually annotated set based on which the annotator later gets suggestions for the most suitable labels potentially reducing the annotation effort.

There are also methods that provide fully automated annotation of the sensor data. It is based on supervised or unsupervised machine learning methods that attempt to automatically segment and/or label the data [21,22]. A potential problem with automated and semi-automated annotation tools is that they capture the behaviour only on the sensors granularity level. In other words, such tools will potentially be unable to capture fine grained activities or properties of the environment that are unobserved by the sensors [23]. This could be a problem for symbolic approaches for activity recognition that aim to reason about the observed behaviour in a causal or semantic manner [9].

To address the above problems, in the following sections, we propose an approach that provides high quality model-based annotation, which allows for reasoning about hidden properties and causal relations in the annotation.

### 2.2. Types of Annotation Based on Underlying Label Structure

In the context of human behaviour recognition, we distinguish between three different types of annotation with respect to the label structure. The first is the annotation of activities where a textual description (or label) is assigned to the executed action [19,24,25,26]. More formally, the objective is to manually assign a label $l_{i}$ to each time step of a time series. This is often done by analysing a separately recorded video log of the executed activities. In case of activity recognition, this annotation sequence is later used for 1. learning models in methods of supervised machine learning, and 2. quantifying the performance of the method under test. These labels are usually called *ground truth*, as they provide a symbolic representation of the true sequence of activities. However, for the finite set $L=\{l_{1}\ldots l_{n}\}$ of labels, there is usually no further information besides the equality relation. Annotations such as take-baking_pan provide a textual description of the executed task that, however, do not contain an underlying semantic structure. There is usually no formal set of constraints that restrict the structure of the label sequences. Typically, nothing prevents an annotator from producing sequences like “put fork to drawer” → “close drawer” → “take knife from drawer”. This is also the most common type of annotation of human behaviour, partially because even the assignment of non-semantic labels to the data is a difficult, time-consuming, and error prone task [19].

The second type of annotation is the plan annotation. It can be divided into goal labelling and plan labelling [27]. The goal labelling is the annotation of each plan with a label of the goal that is achieved [28,29]. In contrast, plan labelling provides annotation not only of the goal, but also of the actions constituting the plan, and of any subgoals occurring in the plan [30]. The latter is, however, a time-consuming and error prone process [27], which explains why the only attempts of such plan annotation are done when executing tasks on a computer (e.g., executing plans in an email program [30]). This is also reflected in activity and plan recognition approaches such as [31,32] that use only synthesised observations, and thus synthesised annotation, in order to recognise the human actions and goals.

The third type of annotation is the semantic annotation [33]. The term comes from the field of the semantic web where it is described as the process and the resulting annotation or metadata consisting of aligning a resource or a part of it with a description of some of its properties and characteristics with respect to a formal conceptual model or ontology [34]. The concept is later adopted in the field of human behaviour annotation, where it describes the annotating of human behaviour with labels that have an underlying semantic structure represented in the form of concepts, properties, and relations between these concepts [35,36]. We call this type of semantic structure an algebraic representation in accordance with the definition provided in [37]. There, an algebraic representation is one where the state of the system is modelled in terms of combinations of operations required to achieve that state.

In difference to the algebraic representation, there exists a model-based representation which provides a model of the system’s state in terms of collection of state variables. Then, the individual operations are defined in terms of their effects on the state of the model [37]. To our knowledge, there have been no attempts to represent the semantic structure of human behaviour annotation in the form of model-based representation. In the next sections, we present an approach to semantic annotation of human behaviour where the underlying semantic structure uses a model-based representation. This representation allows us to provide not only a semantic meaning to the labels, but also to produce plan labels and to reason about the plan’s causal correctness. Furthermore, it gives the state of the world corresponding to each label and allows for tracking how it changes during the plan execution.

## 3. A Novel Approach to Annotating Human Behaviour

In this section, we present an offline manual model-based semantic annotation approach that strives to overcome the drawbacks of the approaches outlined in the previous section. Our approach combines the characteristics of some state-of-the-art approaches and in addition relies on model-based instead of algebraic knowledge representation. The targeted group of activity datasets, which will potentially benefit from this approach, are those describing goal-oriented behaviour. Typical activity recognition experiments such as the CMU-MMAC [38] can be regarded as goal oriented. In them, the participants are instructed to fulfill a task such as food preparation. To ensure comparability of different repetitions, identical experimental setup is chosen for each trial. As a result, the action sequence executed by the participants can be regarded as a plan, leading from the same initial state (as chosen by the experimenter) to a set of goal states (given in the experiment instruction). In the domain of automated planning and scheduling, plan sequences are generated from domain models, where actions are defined by means of preconditions and effects. A plan is then a sequence of actions generated by grounding the action schemas of the domain leading from an initial state to the goal state. In contrast, in our semantic annotation approach, we manually create plans that reflect the participants’ actions, and define a planning domain, which describes the causal connections of the actions to the state of the world. We use an extension of the Planning Domain Definition Language (PDDL), which allows us to reason not only about the executed plan, but also about the state variables and probability of the plan steps given observations [3]. Below, we describe the proposed annotation process, including the definition of the label set L, the label semantics, the manual annotation procedure, and the validation procedure.

**Figure 1:**
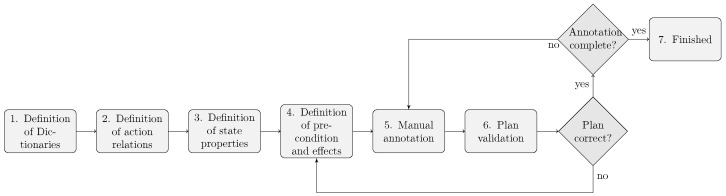
The proposed annotation workflow.

Figure 1 shows the steps in the proposed approach. We illustrate the process with examples from the kitchen domain.

### 3.1. Step One: Action and Entity Dictionary Definition

In the first step, a dictionary of actions and entities (also called objects) is created. The actions have a name representing the action class, and a description of the action class that distinguishes it from the remaining classes. The dictionary also contains the set of all entities observed during the experiment. The dictionary is manually created by domain experts by analysing the video log, which is typically recorded during the experiment. The results of the dictionary definition are the set of action classes and the set of entities manipulated during action execution (see Table 1).

To allow annotators to distinguish between different actions, each action name is accompanied by its definition. If we look at action $a_{1}$ *take*, its definition is *to grab an object*. During the executing of take, the location of the object changes from the initial location to the hand of the person. The action consists of moving the arm to the object, grabbing the object and finally moving the arm back to the body.

### 3.2. Step Two: Definition of Action Relations

In the second step, the action relations have to be defined. For each action, the number and role of involved entities (or objects) are defined. In case of taking, for example, an object and a location, where the object is taken from, are defined. In addition, for each object, possible roles have to be identified. A pot, for example, can be taken, filled, washed, and stirred. The result of this step is the finite set of labels $L=\{l_{1}={\tilde{a}}_{1}^{1},l_{2}={\tilde{a}}_{1}^{2},\ldots ,l_{k}={\tilde{a}}_{n}^{m}\}$, where $\tilde{a}$ defines the syntax of the action relation *a* to be used for the annotation process (see Table 2).

### 3.3. Step Three: Definition of State Properties

As described above, we use a model-based approach (according to [37]) to semantic annotation. We, therefore, have to define the state space by means of state properties. In the third step, a set of state properties is defined as a function of a tuple of entities to an entity of the domain. The state space is then defined by each combination of possible mappings of entity tuples. Finally, the subset of mappings that holds in the initial state (start of the experiment) has to be marked (see Table 3).

### 3.4. Step Four: Definition of Preconditions and Effects

The objective of the fourth step is to define the semantics of the actions. Using the type signature defined in the previous step, action schemes are defined in terms of preconditions and effects. As explained above, we regard the participants’ action sequences as plans. Here, we describe them in PDDL-like syntax [3]. Vanilla PDDL is known from the domain of the automated planning and scheduling, while the extended version we use comes from the domain of sensor-based behaviour analysis and interpretation. The plans and the models can later be analysed with the Computational Causal Behaviour Models (CCBM) validation tool [39]. Besides validating the plans, the tool also provides additional information such as the values of the state variables, the branching factor, or the action probability given a plan and a model.

**Figure 2:**
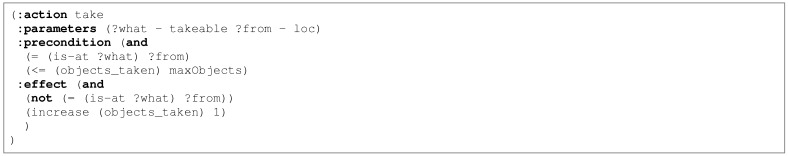
Extract of the action scheme for the take action encodes preconditions and effects in PDDL.

The preconditions and effects for the single action schemes are formed by domain experts. A *take* action, for example, requires an object to be at a certain location and the maximum number of objects not to exceed a certain number. Effects of the take action are that the location of the object is no longer the original location and that the taken objects are increased with one (see Figure 2).

**Figure 3:**
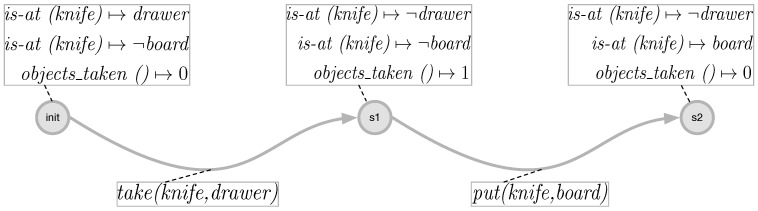
Change in the values of the functions and thus in the state space after the execution of the actions take and put.

An example of how the execution of the actions changes the world state can be seen in Figure 3. It can be seen there that, after the execution of the action take on the objects knife and drawer, the values of the functions change, so that the knife is no longer at a specific location and the taken objects are now one. In the next step, after executing the action put on the object’s knife and board, now the knife is at the location board and the number of objects taken is decreased.

### 3.5. Step Five: Manual Annotation

**Figure 4:**
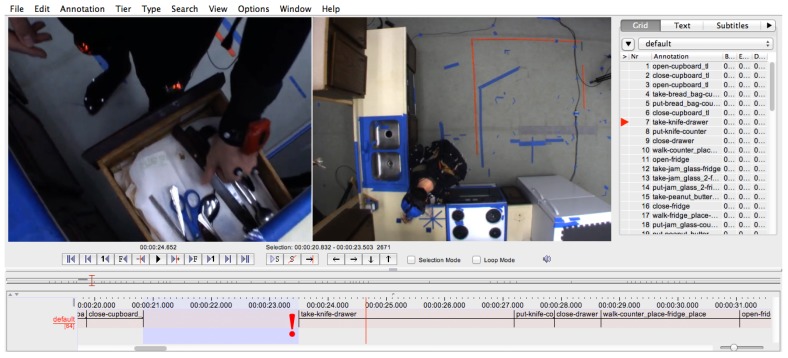
The ELAN tool used to manually create an annotation sequence from the video log. Here, the take-knife-drawer action is being annotated.

Once the dictionary of labels is defined, the manual annotation can be performed. We use the ELAN annotation tool [40] for this step. Here, an annotator has to assign labels from the defined label set to the video sequence. The ELAN annotation tool allows for synchronising several video files and to show them in parallel. Figure 4 shows the ELAN tool during the annotation of a cooking task from the CMU-MMAC dataset.

### 3.6. Step Six: Plan Validation

Since the label sequence produced in the previous step consists of plan operators, the complete sequence can be interpreted as a plan, leading from an initial to a goal state. The objective of the sixth step is to check the causal validity of the label sequence with respect to the planning domain created in the previous step. The CCBM plan validator is used for this task [39]. (Note that generally standard PDDL validators such as VAL [41] can also be used for this step.) If the label sequence does not fulfill the causal constraints of the planning domain, two possible reasons exist: either the planning domain does not correctly reproduce the constraints of the experimental setting or the label sequence is incorrect. An example of error in the constraints would be that the model specifies that the person can take a maximum of three objects, but, in reality, what is observed is that at one point the person has taken four objects. In this case, the validator will return an error although the annotation is correct. In case of an incorrect domain, either the preconditions defined in step four have to be relaxed or the effects have to be revised (e.g., increasing of the maximum allowed objects to be taken). An example of error in the label sequence would be taking the knife from the drawer and then taking it again from the drawer, although the knife is no longer there. In case of an incorrect label sequence, step five (manual annotation) has to be repeated to correct the detected problems. To discover which of the two types of error we have, we first look at the annotation where the error was reported. If we do not find problems in the annotation, then we look into the constraints of the model.

The proposed process has three results: (1) the label sequence, (2) the semantic structure of the labels, and (3) a planning domain, describing the causal relations of the labels.

### 3.7. Improving the Quality of Annotation

It is often the case that two annotators provided with the same codebook produce annotation with a low overlap [42]. This can be explained with the high behaviour variability and with the different interpretation of human behaviour. For example, in the action “take object”, one can interpret the beginning of the action as the point at which the protagonist starts reaching for the object, or the point at which the hand is already holding the object. This deviation in the interpretation reduces the overlapping of labels done by different annotators. To reduce the effect of such discrepancies between annotators, the literature suggests training the annotators, which leads to an increase in the interrater reliability [42]. The idea here is that the annotators are first introduced to the problem and the codebook. They are then trained to recognise the labels from the codebook in the annotated data. After a certain performance level (usually in terms of interrater reliability) is achieved, they can start with the actual annotation of the data. Note that the performance threshold is dependent on the problem domain. An established threshold in the social sciences is overlapping of 80% and above. Another alternative is to train the annotators until the training no longer brings additional improvement in the performance (see Section 4.2.3) Some approaches suggest the training of the annotators by an expert in the problem domain (for example, when training Dementia Care Mapping annotators [43]). That is, the expert teaches the annotators to identify a predefined behaviour and to label it with a label that is predefined for this observation. Different to this approach, we follow a more explorative strategy. There, the annotators have to decide for themselves what the label of the observed behaviour is and later discuss it between themselves and find a consolidated solution. In that manner, the meaning of the observed behaviour is not “enforced” from the system designer or domain expert but is rather consolidated by a group of people. This is similar to the idea of developing an ontology or a folksonomy, where one goal is to collect knowledge accepted by a group and not private of some individual [13,44]. In case the annotators cannot reach an agreement, or are uncertain of how to annotate a given behaviour, the domain expert and system designer are asked for a suitable solution. Furthermore, to ensure the validity of the annotation with respect to the domain, the annotators are asked to document all their decisions and the system designer and the domain expert check that these decisions are valid.

The training phase we propose involves the following steps: 1. the domain expert meets with the annotators and discusses the elements of the dictionary and their presence in an example video log; 2. the annotators separately annotate the same video log with the labels from the dictionary; 3. the annotators compare the two annotations, discuss the differences and decide on a new consolidated annotation of the video log; 4. the annotators document their decisions and the domain expert and the system designer decide on the validity of the decisions; 5. in case, new observations without matching labels are discovered, new labels are introduced to the dictionary after a discussion with the domain expert; and 6. the annotators repeat steps 2, 3, 4, and 5 for the next video log.

In a study conducted in [42], only about 13% of the studies reported the size of the training involved. It was, however, concluded that high intensity training produces significantly better results than low intensity training or no training. For that reason, we performed training as long as the annotators felt comfortable annotating without external help (28% of the data in the concrete annotation scenario).

We applied the proposed annotation process together with the training phase on the CMU Multi-Modal Activity Database [24]. In the next section, we outline the CMU-MMAC and the annotation we created by applying our approach to this dataset.

## 4. Evaluation

### 4.1. Methods and Materials

#### 4.1.1. The CMU-MMAC

The Carnegie Mellon University Multi-Modal Activity Database (CMU-MMAC) provides a dataset of kitchen activities [24]. Several subjects were recorded by multiple sensors (including cameras, accelerometers, and RFIDs) while performing food preparation tasks. A literature research revealed that only a few researchers ever used this dataset. In [45], the activities of twelve subjects were directly reconstructed from the video by use of computer vision. In [46], the cameras and the IMU data were used for temporal classification of seven subjects. We believe that two reasons exist for this publicly available dataset to not be further used in the literature. The first is that activity recognition in the kitchen domain is a very challenging task and the second is that the provided annotation is neither complete nor provides enough information to efficiently train classifiers. In the following section, we briefly describe our annotation for the CMU-MMAC.

##### Overview of the CMU-MMAC

The CMU-MMAC consists of five sub datasets (Brownie, Sandwich, Eggs, Salad, Pizza). Each of them contains recorded sensor data from one food preparation task. The dataset contains data from 55 subjects, where each of them participates in several sub experiments. While executing the assigned task, the subjects were recorded with five cameras and multiple sensors. While the cameras can be used for computer vision based activity recognition [45], the resulting video log is also the base for the dataset annotation. An annotated label sequence for 16 subjects can be downloaded from the CMU-MMAC website (see [38]). Albeit following a grammatical structure of verbs and objects, the label sequence is still missing semantics which, if present, would allow the deriving of context information such as object locations and relations between actions and entities. In the following section, we discuss the annotation of three of the five datasets (Brownie, Sandwich, and Eggs). The annotation and the corresponding models can be found at [47].

##### Complexity of the CMU-MMAC

The sub datasets in the CMU-MMAC describe the preparation of different meals. All meals, however, have similar structure in terms of types of executed actions. On the one hand, this limits the evaluation of the approach as the dataset illustrates only one type of everyday activity (i.e., cooking). On the other hand, the annotation is very fine-grained, which produces high variability in the between person and between datasets’ behaviour. There is mean overlapping of 3.7% between the behaviour of the different participants in the Brownie dataset, 5.1% for the Eggs, and 7.3% for the Sandwich. These results are without considering the durations of the actions, which will produce additional variability between the participants. Furthermore, although the experiment is scripted, the participants show behaviour such as abandoning actions, subroutines, time-sharing, and non-goal oriented actions. For that reason, we believe that the selected dataset adequately illustrates the capabilities of the proposed approach. Furthermore, in Section 5, we discuss the applicability of the approach to other problems from the activities of daily living domain.

#### 4.1.2. Experimental Setup

In order to evaluate the proposed annotation process and the quality of the resulting annotation, we conducted the following experiments: 1. Two domain experts reviewed a subset from the video logs for the Brownie, Eggs, and Sandwich datasets and identified the action classes, entities, action relations, state properties, and precondition-effect rules. 2. Two annotators (Annotator A and Annotator B) independently annotated the three datasets (Brownie, Eggs, and Sandwich). 3. The same two annotators discussed the differences in the annotation after each annotated video log for the first *n* videos of each dataset and prepared a consolidated annotation for 28% of the sequences in the datasets. This number *n* is 12 for the Brownie, 7 for the Eggs, and 6 for the Sandwich dataset.

Based on these annotated sequences, we examined the following hypotheses:

**Hypothesis 1** **(H1).** *Following the proposed annotation process provides a high quality annotation*.

**Hypothesis 2** **(H2).** *Training the annotators improves the quality of the annotation*.

**Hypothesis 3** **(H3).** *We can directly reason about hidden properties and behavioural routines based on the annotation and the underlying model*.

To test H1, we calculated the interrater reliability between Annotator A and Annotator B for all video logs in the three datasets (90 video logs).

To test H2, we investigated whether the interrater reliability increases with the training of the annotators. The interrater reliability was calculated for the ground labels, not for the classes (in other words, we calculate the overlapping between the whole label “take-bowl-cupboard” and not only for the action class “take”). The interrater reliability was calculated in terms of agreement ($IR_{a}$), Cohen’s κ ($IR_{\kappa }$), and Krippendorff’s α ($IR_{\alpha }$). We chose the above measures as they are the most frequently used measures for interrater reliability as reported in [42].

To test H3, we investigated whether we can identify behavioural routines and reason about semantic properties from the annotated datasets. To do that, we generated a directed graph representing the causally correct behavioural routines for each type of experiment. We also investigated the ability to identify different initial conditions based on the annotation. Furthermore, we showed that it is possible to reason about the state of a given object in the experiment based on the annotation. Finally, we derived a “recipe” based on the annotated manipulation with actions.

### 4.2. Results

#### 4.2.1. Semantic Annotation for the CMU-MMAC

To define the label set, two domain experts reviewed a subset from the video logs and identified 13 action classes (11 for the Brownie, 12 for the Eggs, and 12 for the Sandwich). Table 4 shows the action classes for the three datasets.

The action definitions created in this step later enable different annotators to choose the same label for identical actions. In this step, the domain experts also identified the entities (30 for the Sandwich dataset, 44 for the Brownies, and 43 for the Eggs). From these dictionaries, in step two, a discussion about the type signature and possible instantiations took place (119 unique labels were identified for the Sandwich dataset, 187 for the Brownies, and 179 for the Eggs; see Table 2 for examples). Step three, definition of state properties, revealed 13 state properties (see Table 3). The next three steps were executed by two annotators until all datasets were annotated without gaps and all annotation sequences were shown to be valid plans.

Table 5 shows an extract of the annotated plan for subject S09 from the Brownie dataset.

The resulting annotation consists of 90 action sequences. Interestingly, while annotating, we noticed that the experimenter changed the settings during the experiments’ recording. In all sub-experiments, it can be seen that, before recording subject 28, some objects were relocated in different cupboards. Our annotation is publicly available to enable other researchers to address the activity recognition problem with the CMU-MMAC dataset. The complete annotation and the corresponding models can be accessed at [47]. We believe that the annotation will contribute to the development of both data- and knowledge-driven approaches for activity recognition, as the CMU-MMAC dataset is currently lacking complete or even validated annotation. This limits its usefulness for the activity recognition community.

#### 4.2.2. Results for H1

**Figure 5:**
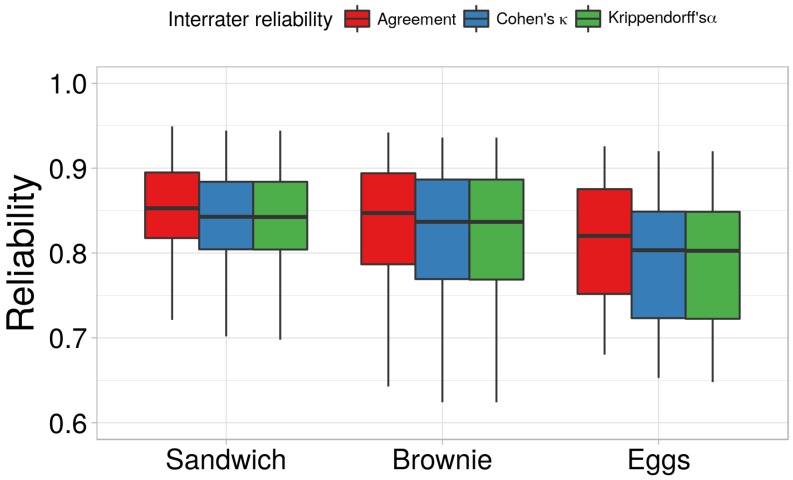
The median interrater reliability for the three datasets (in terms of Cohen’s κ) and the deviation from this median.

To address H1, we computed the interrater reliability between Annotator A and Annotator B. The results can be seen in Figure 5. It can be seen that the annotators reached a median agreement of 0.84 for the Brownie, 0.81 for the Eggs, and 0.84 for the Sandwich. Similarly, the Cohen’s κ and the Krippendorff’s α had a median of 0.83 for the Brownie, 0.80 for the Eggs, and 0.83 for the Sandwich. A Cohen’s κ between 0.41—0.60 means moderate agreement, between 0.61—0.80 means substantial agreement, and above 0.81 indicates almost perfect agreement [48].

Similarly, data that has Krippendorff’s α above 0.80 is considered to be reliable to draw conclusions. In other words, the average interrater reliability between the two annotators is between substantial and almost perfect. This also indicates that the proposed annotation process not only provides semantic annotation, it also ensures that the annotators produce high quality annotation. Consequently, hypothesis H1 was accepted.

**Figure 6:**
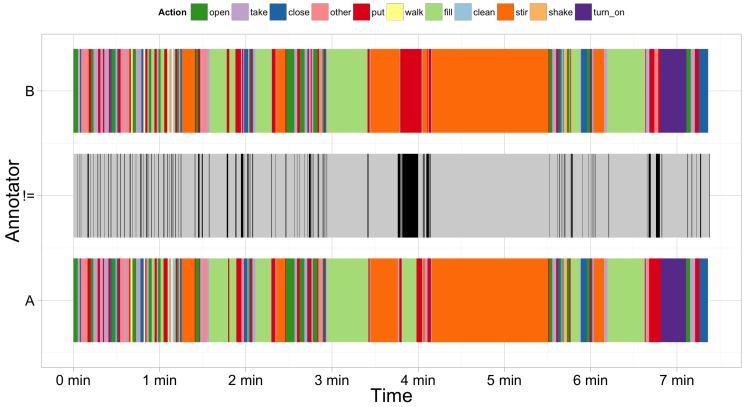
Comparison between the annotation of a video log of Annotator A (bottom) and Annotator B (top) from the “Brownie” dataset for subject 9. The different colours indicate different action classes. The plot in the middle illustrates the differences between both annotators (in black).

Figure 6 shows the annotated by Annotators A and B classes and the places where they differ. It can be seen that the difference is mainly caused by slight shifts in the start and end time of the actions. This indicates that the problematic places during annotation of fine-grained actions are in determining the start and end of the action.

#### 4.2.3. Results for H2

**Figure 7:**
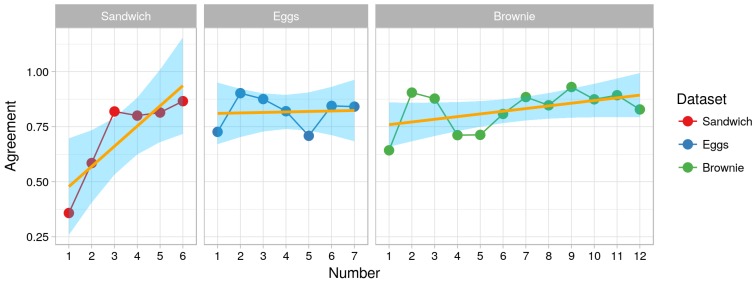
Learning curve for the first *n* videos. The points illustrate the percentage of interrater agreement for one dataset. The points are connected to increase perceivability. The orange line illustrates the increase of reliability due to learning.

To test H2, we investigated whether the training had an impact on the interrater reliability. We calculated the difference in the interrater reliability between each newly annotated video log and the previous one during the training phase. The “Brownie” dataset has mean positive difference of about 2% while “Sandwich” has a mean difference of about 10%. This means that on average there was an improvement of 2% (respectively 10%) in the interrater reliability during the training phase. On the other hand, the “Eggs” dataset shows a negative difference of 1%, which indicates that on average no improvement in interrater reliability was observed during the training phase. A negative difference between some datasets was also observed. These indicate decrease in the interrater reliability after training was performed (with maximum of about 2%). Such decrease can be explained with encountering of new situations in the dataset or with different interpretation of a given action. However, a decrease of 2% does not significantly reduce the quality of the annotation. Figure 7 illustrates the interrater agreement for the datasets selected for the training phase. The orange line shows a linear model that was fitted to predict the interrater reliability from the dataset number. It can be seen that the effect of the training phase was not negative for all datasets. For two datasets (Brownie and Sandwich), an increasing trend can be seen.

To better understand the change in the interrater reliability, we look into the agreement ($IR_{a}$) between the annotators of the first six annotations of the “Sandwich” dataset (Figure 7).

The interrater reliability between the first and the second annotated video increases by 23%. The same applies for the interrater reliability between the second and the third annotated video. At that point, the interrater reliability has reached about 81% overlapping (Cohen’s κ of 0.8), which indicates almost perfect overlapping. After that, there is a mean difference of about 1%. On average, the overlapping between the two annotators stays around 80% (or mean Cohen’s κ of 0.78) even after the training phase. This indicates that the learning phase improves the agreement between annotators, thus the quality of the produced annotation (hence we accept H2).

Interestingly enough, the results show that one needs a relatively small training phase, two videos in case of the “Sandwich” and one video for “Brownie” and “Eggs”, to produce results with almost perfect overlapping of about 80% between annotators. This contradicts the assumption that we need high intensity training to produce high quality annotation (as suggested in [42]). It also shows that using our approach for semantic annotation ensures a high quality annotation without the need of intensive training of the annotators.

#### 4.2.4. Results for H3

To investigate H3, we show that the annotation provides us with various pieces of information about the state of the world and the behaviour patterns of the study participants.

**Figure 8:**
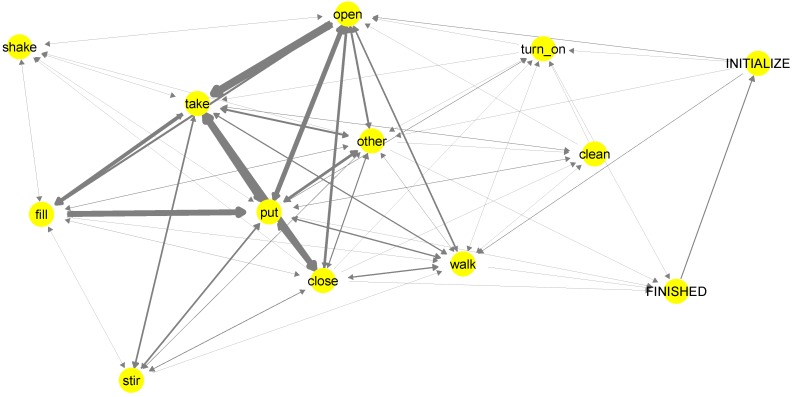
Graph representing the causally correct transitions between action classes. Thicker arrows indicate more probable transitions. Note that, besides the action classes from Table 4, we also have initial and finish actions marking the initial and goal conditions of the model.

As the produced semantic annotation has an underlying world model, we are able to derive behavioural routines out of the annotation. Figure 8 shows a first order Markov model for the “Brownie” dataset. The model represents the behaviour for all participants. The thicker the arrow, the more often this transition was observed in the annotation (i.e., the corresponding sequence of actions is more probable). Different to models generated from non-semantic annotation, in our case, the underlying semantic structure ensures that the Markov model is causally correct. In other words, there are no causally impossible transitions between actions (e.g., teleporting from place to place or executing tasks on objects that are not available at the current location).

Note that the model presented in Figure 8 is based only on the action classes. The same type of model can also be generated for the ground actions, containing contextual information such as objects and locations. The model can be considered as a sub-model of the proposed model, where the proposed model additionally contains various pieces of information about the annotated contextual elements, goals and causes of behaviour, as well as the change of these properties through the execution of actions.

**Figure 9:**
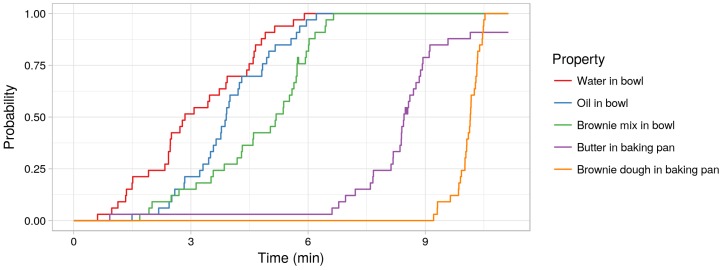
Example of a reasoning about the executed sequence. The probability of particular environment properties is given per time step. Furthermore, from the probability of the butter being in the baking pan, it can be seen that some participants forgot to put butter in the baking pan.

Another advantage of the produced annotation is that we are able to reason about environmental properties based on the annotation. Figure 9 shows an example of reasoning about some of the ingredients needed to prepare brownies and the change of their probability over time. The probability is calculated based on all observed instances of these properties for all runs of the “Brownie” dataset. It can be seen that there is a well-defined sequence of changes of the environmental properties. First, the water is poured into the bowl, then the oil, then the brownie mix. After the dough is ready (which is not plotted here), the baking pan is oiled with butter and the dough is placed in the baking pan. Also interesting in the plot is that the participants sometimes forgot to oil the baking pan (the probability of the butter being in the baking pan does not reach 1). In that manner, we are able to reason about the typical “recipe” as well as about deviations in this recipe and even about possible causes of observed deviations.

**Figure 10:**
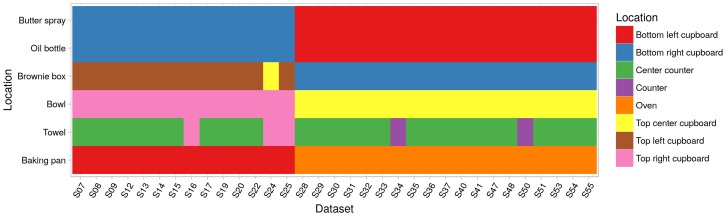
The different initial locations of some objects. It can be seen that, from S28, the locations of most objects were changed at the beginning of the experiment. The towel, for instance, was placed at the center of the counter except for five datasets where the towel was either on the other counter or a cupboard.

The approach and the generated annotation also allows for reasoning about changes in the initial and goal states of the experiment. For example, Figure 10 shows the initial locations for several objects. It is interesting to note that the initial setup suddenly changed after run S25.

This is something that was not described in the provided by CMU documentation. It is, however, an important piece of information, especially when developing or training models that reason about the sequences of actions and the context, not only about the action label.

**Figure 11:**
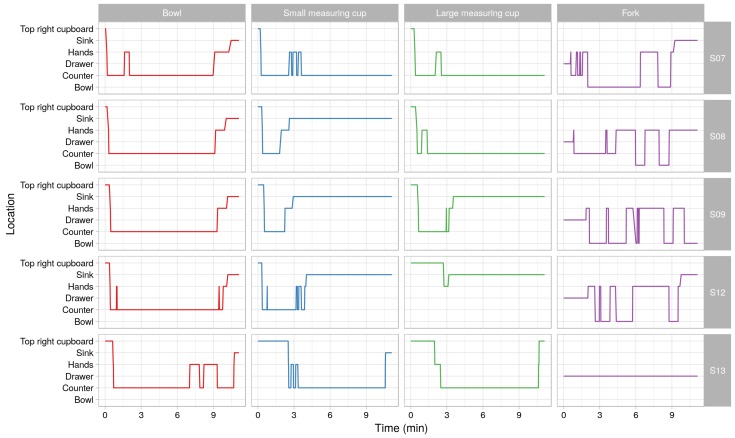
The location of different objects over time for different datasets.

Finally, it is possible to reason about the change of location of separate objects throughout a given run. Figure 11 shows the change of location of four objects in five different runs. It can be seen that, although there are obvious patterns in the change of locations, the participants also had their personal way of executing the experiment. This led to between person variability of the objects’ locations throughout the experiment.

## 5. Discussion

In this work, we proposed an approach for semantic annotation of sensor datasets, which was evaluated in the CMU-MMAC dataset. This dataset consists of different cooking activities, which, however, have a very similar semantic structure. Apart from the CMU-MMAC dataset, we have, however, applied the approach to several different datasets describing daily activities. In [49], we have produced both coarse- and fine-grained annotation for a variety of kitchen tasks in real settings. The coarse-grained annotation is on a higher granularity level than the annotation of the CMU-MMAC dataset and it was produced in one week with a relatively small effort. The fine-grained annotation is on the granularity level of the CMU-MMAC annotation, but it has much higher complexity and variability as the data was recorded in real settings. It took a trained annotator about three months to complete this annotation.

Apart from the cooking domain, we are currently annotating the behaviour of people during assembly tasks (e.g., assembling shelves) and of people in warehouses. A lot of effort goes in the initial identification of the relevant world elements that are to be modelled and the training of the annotator for the new type of task. However, after this initial period, the effort of annotating the dataset and adding any new elements to the model is relatively low.

We have also followed the approach when annotating the behaviour of people with dementia during their outdoor mobility [50]. People with dementia do act goal-oriented. Sometimes, they forget things (which increases with the progression of the disease), which results in adding additional variability and non-goal oriented actions to the behaviour. Using causal modelling for annotating the behaviour allows for identifying non-goal oriented actions as well as problems in the exhibited behaviour. This could later be used to detect problematic behaviour and automatically assist the person in achieving their goal. As modelling the behaviour of people with dementia in their outdoor mobility is a very complex domain in terms of hidden factors influencing the behaviour, a large effort was invested in gathering the domain knowledge needed to interpret the observed behaviour and the psychological, environmental, and person-specific reasons behind a given behaviour. This effort, however, does not reflect the complexity of the specific annotation approach, as any context-aware system addressing this problem domain would need to first gather the domain knowledge.

We have not tested the approach outside the domain of human behaviour annotation. We, however, believe that the approach is applicable to any dynamic system that acts under the assumption of causality.

Another problem is that, to produce high quality annotation, it takes a lot of time and effort not only for the annotators, but also for the annotation designers who have to manually build the semantic model used for validating the annotation. To address this problem, it is possible to extend the approach so that it automatically generates the underlying semantic model needed for validating the annotation. This can be done by adapting the idea of learning models of human behaviour from textual instructions proposed in works such as [51,52,53,54]. One can use this to automatically generate the underlying semantic model from detailed textual descriptions of the experiment.

## 6. Conclusions

In this work, we presented a novel approach to manual semantic annotation. The approach allows the usage of a rich label set that includes semantic meaning and relations between actions, entities and context information. Additionally, we provide a state space that evolves during the execution of the annotated plan sequences. In contrast to typical annotation processes, our annotation approach allows for further reasoning about the state of the world by interpreting the annotated label sequence as grounded plan operators. It is, for example, easy to infer the location of objects involved, without any explicit statement about the objects’ location.

To validate our approach, we annotated the “Brownie”, “Eggs”, and “Sandwich” trials from the CMU-MMAC dataset. In the original annotation, only 16 out of 90 sequences are annotated. We now provide a uniform annotation for all 90 sequences including a semantic meaning of the labels. To enable other researchers to participate in the CMU grand challenge, we make the complete annotation publicly available. Furthermore, we evaluated the quality of the produced annotation by comparing the annotation of two annotators. The results showed that the annotators were able to produce labelled sequences with almost perfect overlapping (Cohen’s κ of about 0.8). This stands to show that the approach provides high quality semantic annotation, which the ubiquitous computing community can use to further the research in activity, plan, and context recognition.

In the future, we intend to investigate the automation of the approach as discussed in Section 5. We will compare the automated approach to our manual process in order to determine whether it improves the time and effort required for producing semantic annotation. We will also investigate whether such automated approach reduces the quality of the produced annotation.

## Figures and Tables

**Table 1:** Result of step 1: A dictionary of actions and entities.

	Action		Entity
$a_{1}$	take	$e_{1}$	knife
$a_{2}$	put	$e_{2}$	drawer
$a_{3}$	walk	$e_{3}$	counter
	...		...
$a_{n}$	stir	$e_{m}$	pepper

**Table 2:** Result of step 2: The table lists the type signature and each possible instantiation for the set of actions identified in the previous step. Here “what: takable” denotes any entity from type “takable”, similarly “from: location” denotes any entity of type location.

$a_{1}$	***take* (*what*:takeable, *from*:location)**
${\tilde{a}}_{1}^{1}$	take (knife, drawer)
${\tilde{a}}_{1}^{2}$	take (knife, board)
	...
${\tilde{a}}_{2}$	***put* (*what*:takeable, *to*:location)**
${\tilde{a}}_{2}^{1}$	put (knife, drawer)
${\tilde{a}}_{2}^{2}$	put (knife, board)
	...

**Table 3:** Result of step 3: A list of functions with type signatures and their instantiations. A * in the last column means that the defined function holds in the initial state.

$f_{1}$	***is-at* (*what*: takeable) → location**	
$f_{1}^{1}$	is-at (knife) ↦ drawer	*
$f_{1}^{2}$	is-at (knife) ↦ board	
	...	
$f_{2}$	***objects_taken* () → number**	
$f_{2}^{1}$	objects_taken () ↦ 0	*
$f_{2}^{2}$	objects_taken () ↦ 1	
	...	

**Table 4:** Action classes for the three datasets.

Dataset	Action Classes
Brownie	open, close, take, put, walk, turn on, fill, clean, stir, shake, other
Eggs	open, close, take, put, walk, turn on, fill, clean, stir, shake, other,
	turn off
Sandwich	open, close, take, put, walk, turn on, fill, clean, stir, shake, other, cut

**Table 5:** Extract of the annotated plan from the Brownie dataset of subject S09. The overall number of actions is 142.

	Start Time	Action
1	00:00.000	open-cupboard_tl
2	00:03.198	take-brownie_box-cupboard_tl
3	00:04.616	close-cupboard_tl
4	00:05.456	other
5	00:10.735	put-brownie_box-counter
6	00:12.006	open-cupboard_tr
7	00:13.941	take-bowl-cupboard_tr
8	00:17.044	put-bowl-counter
9	00:18.522	take-measuring_cup_s-cupboard_tr
10	00:20.540	put-measuring_cup_s-counter
11	00:21.571	take-measuring_cup_l-cupboard_tr
12	00:24.704	put-measuring_cup_l-counter
13	00:25.682	close-cupboard_tr
14	00:26.708	open-cupboard_br
15	00:29.379	take-oil_bottle-cupboard_br
16	00:30.412	close-cupboard_br
17	00:31.313	put-oil_bottle-counter
18	00:32.758	other
19	00:35.519	take-brownie_box-counter
20	00:36.784	other
21	00:39.069	put-brownie_box-counter
22	00:40.044	walk-counter_place-fridge_place
23	00:41.175	open-fridge
24	00:42.547	open-egg_box
25	00:43.660	take-1-egg_shell-egg_box
26	00:45.432	take-1-egg_shell-egg_box
27	00:46.697	close-egg_box
28	00:47.296	close-fridge
29	00:48.724	walk-fridge_place-counter_place
30	00:50.082	put-2-egg_shell-counter
31	00:50.946	take-1-egg_shell-counter
32	00:52.502	open-egg_shell
33	00:54.224	fill-egg-open_egg_shell-bowl
34	00:56.411	put-1-empty_egg_shell-sink
35	00:57.910	take-1-egg_shell-counter
36	00:58.976	take-1-egg_shell-counter
37	00:58.980	open-egg_shell
38	01:00.668	fill-egg-open_egg_shell-bowl
39	01:03.167	put-1-empty_egg_shell-sink
40	01:05.817	walk-counter_place-fridge_place

## Abbreviations

The following abbreviations are used in this manuscript:

CCBM	Computational Causal Behaviour Models
CMU	Carnegie Mellon University
CMU-MMAC	Carnegie Mellon University Multi-Model Activity Database
PDDL	Planning Domain Definition Language
